# NINE YEAR CHANGES IN PREVALENCE OF COGNITIVE IMPAIRMENT IN THE CZECH REPUBLIC

**DOI:** 10.1093/geroni/igz038.3178

**Published:** 2019-11-08

**Authors:** Dominika Seblova, Marie Kuklova, Miloslav Kopecek, Pavla Cermakova, Carol Brayne, Vendula Machu

**Affiliations:** 1 Columbia University, Taub Institute for Research in Alzheimer’s Disease and the Aging Brain - The Gertrude H. Sergievsky Center, New York, New York, United States; 2 Department of Public Health and Primary Care, Cambridge Institute of Public Health, University of Cambridge, Cambridge, United Kingdom; 3 National Institute of Mental Health, Klecany, Czech Republic

## Abstract

Studies from North America and Western Europe suggest stable or declining trends in impaired cognition. Nevertheless, data on changes in cognitive health from Central and Eastern Europe are largely lacking. Therefore, we aimed to examine changes in the age-specific prevalence of cognitive impairment in the Czech Republic, a country in Central Europe. To this aim we used two samples from the population-based Czech Survey on Health, Ageing and Retirement in Europe (SHARE). Age-specific prevalence of cognitive impairment (defined based on scores in verbal fluency, immediate recall, delayed recall and temporal orientation) was compared between participants in wave 2 (2006/2007; n=1,107) and wave 6 (2015; n=3,104). Logistic regression was used to estimate the association between wave and cognitive impairment, step-wise adjusting for sociodemographic and clinical characteristics. Multiple sensitivity analyses, focusing on alternative operationalisations of relative cognitive impairment, impact of missing cognitive data and survival bias, were carried out. The most conservative estimate suggested that the age-specific prevalence of cognitive impairment declined by one fifth, from 11% in 2006/2007 to 9% in 2015. Decline was observed in all sensitivity analyses. Multivariate decomposition for nonlinear models was used to examine which predictors explain the change in prevalence. Reduction in physical inactivity, control of high blood cholesterol and increases in length of education were the main predictors contributing to decline in cognitive impairment. In conclusion, our findings are in line with those found in North America and Western Europe even though countries in Central and Eastern Europe, including Czech Republic, have poorer risk profiles.

